# Biofortification of oilseed *Brassica juncea* with the anti-cancer compound glucoraphanin by suppressing *GSL-ALK* gene family

**DOI:** 10.1038/srep18005

**Published:** 2015-12-10

**Authors:** Rehna Augustine, Naveen C. Bisht

**Affiliations:** 1 National Institute of Plant Genome Research (NIPGR), Aruna Asaf Ali Marg, New Delhi 110067, INDIA

## Abstract

Glucosinolates are amino acids derived secondary metabolites, invariably present in Brassicales, which have huge health and agricultural benefits. Sulphoraphane, the breakdown product of glucosinolate glucoraphanin is known to posses anti-cancer properties. AOP (2-oxoglutarate-dependent dioxygenases) or GSL-ALK enzyme catalyzes the conversion of desirable glucoraphanin to deleterious gluconapin and progoitrin, which are present in very high amounts in most of the cultivable *Brassica* species including *Brassica juncea*. In this study we showed that *B. juncea* encodes four functional homologs of *GSL-ALK* gene and constitutive silencing of *GSL-ALK* homologs resulted in accumulation of glucoraphanin up to 43.11 μmoles g^−1^ DW in the seeds with a concomitant reduction in the anti-nutritional glucosinolates. Glucoraphanin content was found remarkably high in leaves as well as sprouts of the transgenic lines. Transcript quantification of high glucoraphanin lines confirmed significant down-regulation of *GSL-ALK* homologs. Growth and other seed quality parameters of the transgenic lines did not show drastic difference, compared to the untransformed control. High glucoraphanin lines also showed higher resistance towards stem rot pathogen *Sclerotinia sclerotiorum*. Our results suggest that metabolic engineering of *GSL-ALK* has huge potential for enriching glucoraphanin content, and improve the oil quality and vegetable value of *Brassica* crops.

Glucosinolates (GSL) are nitrogen and sulfur rich secondary metabolites mostly present in the order Brassicales. Upon tissue damage, these compounds are hydrolysed by β-thioglucoside glucohydrolases called myriosinases into various biologically active compounds like isothiocyanates (ITC) and nitriles^1^. Inherent as defense compounds, glucosinolates protect Brassicaceae plants against a wide range of herbivores, insects and pathogenic invaders^2^. Glucosinolate breakdown products also impart aroma and flavour to Brassicales which finds its utilization in culinary purposes from time immemorial. Sulphoraphane (4MSOB-ITC), the isothiocyanate derived from 4-methylsulfinylalkyl glucosinolate (glucoraphanin, GRA) is known to induce phase-II detoxification enzymes during tumour progression and thus act as anti-carcinogenic agents^3^. However degradation products like oxazolidine-2-thione derived from 2-hydroxy-3-butenyl (progoitrin, PRO) glucosinolate have goitrogenic effect on livestock^4^,^5^.

Glucoraphanin is the most widely investigated glucosinolate in the area of health benefits. Healing properties of sulphoraphane, in breast, cervical, prostrate, colon and stomach cancer are well established. Studies show that diet rich in sulphoraphane can fight against *Helicobacter pylori* causing stomach ulcers. Sulphoraphane can also protect against cystic fibrosis, aging, rhinitis, arthritis, asthma and other lung disorders. Hence regular consumption of cruciferous vegetables is highly recommended^3^,^6^,^7^,^8^,^9^,^10^. Among the cruciferous vegetables broccoli contains highest amount of glucoraphanin. Other members of *Brassica oleracea* like chinese kale, cabbage and brussels sprout also possess significant amounts of glucoraphanin. However, numerous *Brassica* cultivars grown for vegetable or oil purpose contain less or negligible amount of glucoraphanin. *B. juncea* is an important oilseed crop cultivated worldwide in central and south Asia, Europe and North America, northern Africa and China. Even though cultivated mainly as an oilseed, leaves of the young plants are consumed as vegetable as well. In general, 3-butenyl (gluconapin, GNA), 2-propenyl (sinigrin, SIN) and 4-pentenyl (glucobrassicin, GBN) glucosinolates are the major aliphatic glucosinolates present in *B. juncea*^11^. The presence of high amounts of these glucosinolates in *B. juncea* is anti-nutritional in nature^12^. Hence metabolic engineering of *B. juncea* for enrichment of desirable glucosinolate glucoraphanin and reducing the anti-nutritional glucosinolates seems highly essential to improve the food and feed value of this crop.

Glucosinolate biosynthesis occurs from amino acid precursors through three major processes *viz.*, chain elongation, core structure formation and side chain modifications (Fig. 1). Glucosinolates are classified as aliphatic, aromatic and indolic glucosinolates based on their precursor amino acids^1^,^13^. Aliphatic glucosinolates can be further classified into 3C, 4C and 5C based on their side chain length, or methylthioalkyl, methylsulfinyl alkyl, alkenyl and hydroxyalkenyl based on the side chain structure^14^. The vast glucosinolates diversity is attributed to the side chain elongation and side chain modification reactions. *GSL-ELONG* gene locus encoding methylthioalkyl malate synthases (MAM) control the initial chain elongation reactions^15^. A subclade of flavin-monooxygenase (*FMO*_*GS-OX1-5*_) catalyzes the conversion of methylthioalkyl glucosinolates into methylsulfinylalkyl glucosinolates^16^,^17^. Two α-ketoglutarate-dependent dioxygenases AOP2 and AOP3 are demonstrated to control the conversion of methylsulfinylalkyl to alkenyl- and hydroxyalkenyl glucosinolates, respectively^18^,^19^.

Accumulation of glucoraphanin is governed by the *GSL-AOP* locus which contains the *GSL-ALK (AOP2)* and *GSL-OHP (AOP3*) locus. Non functional gene product of this loci results in the accumulation of glucoraphanin^20^,^21^. In *Arabidopsis thaliana* ecotype Columbia, *AOP2* gene was shown to be marginally expressed which results in accumulation of glucoraphanin in this accession^22^. In broccoli, non functional *GSL-ALK* homolog has been identified which is associated with high glucoraphanin accumulation. In *B. rapa* three *AOP2* genes identified were found to be functional which explains the reason for the absence of glucoraphanin in *B. rapa* and other related *Brassica* species^14^,^23^,^24^. The high amount of gluconapin and absence of glucoraphanin in *B. juncea* necessitates the characterization and manipulation of these gene homologs in *B. juncea*. Since *B. juncea* is an allotetraploid, where gene duplication and redundancy make genetic manipulation, the only alternative to achieve high glucoraphanin accumulation is through silencing of the *GSL-ALK* gene family. Hence the study is undertaken to isolate all homologs of the *GSL-ALK* gene from *B. juncea* and its subsequent utilization to develop high glucoraphanin lines which can improve the food and feed value of this oilseed crop as a potential source of anti-cancer compounds.

## Results

### Isolation and sequence analysis of *GSL-ALK* gene homologs from *B. juncea*

The full-length genomic sequences of the *GSL-ALK* genes were isolated from *B. juncea* high glucosinolate cultivar Varuna using degenerate primers in our previous study^25^. Based on the genomic sequences, primers were designed and full-length cDNA sequences were isolated. Four full-length coding sequences were identified from *B. juncea*. Contributor sub-genomes of the four homologs were ascertained by simultaneous isolation and sequence comparison of the *GSL-ALK* orthologs from both *B. rapa* (AA) and *B. nigra* (BB), the progenitor sub-genomes of *B. juncea* (AABB). The homologs were named as *BjuA.GSL-ALK-1, BjuA.GSL-ALK-2, BjuB.GSL-ALK-1* and *BjuB.GSL-ALK-2* (A stands for A-sub-genome and B-stands for B-sub-genome). Comparison of coding sequences with the reported genomic sequences of *GSL-ALK* gene homologs^25^ showed the presence of three exons intervened by two introns (Supplementary Table S1). The coding sequences of *BjuA.GSL-ALK-1* and *BjuB.GSL-ALK-1* genes were found to be 1323 bp whereas the CDS of *BjuA.GSL-ALK-2* and *BjuB.GSL-ALK-2* was 1320 bp long with a deletion of single codon. The coding sequences of *BjuGSL-ALK* genes shared around 75% sequence identity with *A. thaliana AOP2* (Cvi) gene and >90% identity among themselves (Supplementary Table S2). The deduced amino acid sequence also showed an identity percentage of 87–99% among the four *B. juncea* GSL-ALK homologs (Supplementary Table S3, Supplementary Fig. S1). The A-sub-genome specific homologs were more similar to each other and similarly the B-sub-genome homologs.

**Figure 2:**
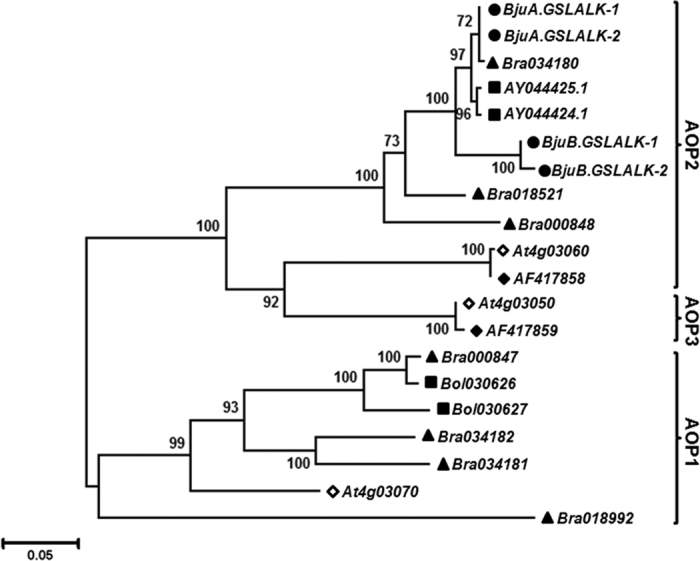
Phylogenetic relationship of *GSL-ALK* (AOP) genes. Phylogenetic analysis of *GSL-ALK* cDNA with the other *AOP* sequences from *Arabidopsis* and related *Brassica* species was performed. The *AOP* like genes from *A. thaliana* ecotype Columbia (◇), *A. thaliana* ecotype Cvi (◆), *B. rapa* (▴), *B. oleraceae* (▄) and *B. juncea* (●) were used to construct the tree. The evolutionary history was inferred by using the Maximum Likelihood method in MEGA5^45^. The percentage of trees in which the associated taxa clustered together is shown next to the branches. The tree is drawn to scale, with branch lengths measured in the number of substitutions per site.

To illustrate the evolutionary relationships among the *GSL-ALK* gene homologs, phylogenetic tree was constructed using *AOP* sequences from *A. thaliana* and *Brassica* species (Fig. 2). On the maximum likelihood tree, the *AOP1, AOP2* and *AOP3* genes resolved into two distinct clusters, in which *AOP2* and *AOP3* were clustered together. The four *BjuGSL-ALK* genes grouped nicely in the *AOP2* clade along with other *Brassica* homologs. Within the *AOP2* clade, the two A-sub-genome specific homologs were grouped together with that of *B. rapa* (Bra034180) and *B. oleracea* (AY044424.1, AY044425.1) sequences, whereas the B-sub-genome specific homologs were grouped separately into another subgroup with high bootstrap support. The phylogenetic tree clearly demonstrated that all four *GSL-ALK* genes isolated from *B. juncea* were *AOP2* type and were evolutionary conserved.

The divergence time analysis of *AOP* genes based on the synonymous base substitution (Ks) values between *Brassica* and *Arabidopsis* (Cvi) genes indicated that both *BjuGSL-ALK* A- and B- sub-genome specific genes diverged somewhere around 14.8–16.0 MYA (Supplementary Table S4), similar to that observed for *B. rapa* (Bra034180, Bra018521) and *B. olereaceae* (AY044424.1, AY044425.1) *GSL-ALK* gene orthologs. Thus, the multiple *BjuGSL-ALK* genes seem to have evolved as a result of whole genome triplication events which is hypothesized to have occurred somewhere around 13–17 MYA in ancestral *Brassica* prototype^26^ followed by an allopolyploidization of two simpler *Brassica* genomes.

### Knock-down of *GSL-ALK* gene provides enrichment of glucoraphanin in transgenic *B. juncea*

**Figure 3:**
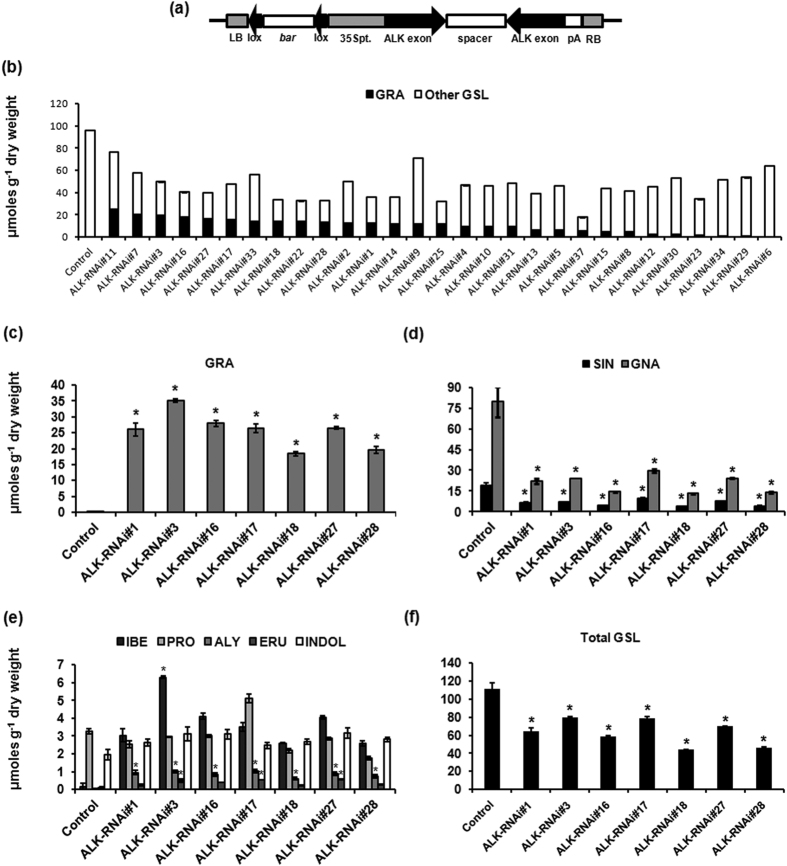
Development of ALK-RNAi transgenic lines of *B. juncea*. (**a**) The T-DNA map of hairpin RNAi cassette of *GSL-ALK* gene. A 325 bp coding region of the *GSL-ALK* gene was cloned in sense and antisense orientations on both sides of a spacer intron, to facilitate the formation of a hairpin structure. The plant selection marker gene *bar* was cloned within the *lox P* site of bacterial *cre-lox P* recombination system. (**b**) Content of glucoraphanin and the other glucosinolates (GSL) of each line in T1 generation seeds, represented as single data point. (**c**) Glucoraphanin (GRA) content in four day old sprouts of ALK-RNAi lines of *B. juncea*. (**d**) The major glucosinolates sinigrin (SIN) and gluconapin (GNA). (**e**) Other glucosinolates, glucoiberin (IBE), progoitrin (PRO), glucoalyssin (ALY), glucoerucin (ERU) and indolic glucosinolates. (**f**) Total glucosinolates. Glucosinolate content of ALK-RNAi lines was estimated by HPLC (in μmoles g^−1^ DW). Three independent experiments were carried out and the average values are represented along with their standard errors. Asterisks on the top of bars indicate significant differences in glucosinolate content compared to the control, wild-type Varuna at *P* < 0.05, in Fishers LSD test determined by ANOVA.

For knock-down of *GSL-ALK* gene family, intron spliced hairpin RNAi (ihpRNAi) construct (ALK-RNAi) was developed, which consisted of a conserved 325 bp region from third exon of *GSL-ALK* gene homologs. The conserved knock-down cassette was driven by the CaMV35S promoter to achieve constitutive down regulation of the four *GSL-ALK* gene homologs in all the tissue types (Fig. 3a). The transformation cassette was introduced into high glucosinolate Indian cultivar of *B. juncea* (cv. Varuna having a total seed glucosinolate content of ca. 107.52 μmoles g^−1^ DW) through *Agrobacterium* mediated genetic transformation. A total of 29 primary transgenic (T0) lines were generated. The transgenic lines were maintained by selfing under the containment field conditions and analyzed for glucosinolate phenotype.

Transgenic lines developed were analyzed by HPLC to estimate content of glucoraphanin and other glucosinolates in T1 generation. The total seed glucosinolate content in the ALK-RNAi transgenic lines ranged from 17.65–76.30 μmoles g^−1^ DW and the glucoraphanin content ranged from 0–24.71 μmoles g^−1^ DW suggesting variable degree of gene silencing efficiency (Fig. 3b; Supplementary Table S5). More than 93% of the transgenic lines showed enhanced accumulation of glucoraphanin (GRA) compared to that of wild-type Varuna plants. Along with the accumulation of glucoraphanin, a concomitant reduction in major glucosinolates was observed, resulting in overall reduction in total glucosinolate content. For instance, gluconapin (GNA), the most predominant and undesirable aliphatic glucosinolate was reduced to levels as low as 7.98 μmoles g^−1^ DW in the transgenic lines compared to 93.55 μmoles g^−1^ DW of wild-type level. Similarly sinigrin (SIN), the second major seed glucosinolate was also reduced to 1.94 μmoles g^−1^ DW in the transgenic lines whereas wild-type plants had ca. 21.08 μmoles g^−1^ DW (Supplementary Table S5). 4-Methylthiobutyl (Glucoerucin, ERU), the precursor glucosinolate for glucoraphanin, which is otherwise marginally present in wild-type plants was increased up to 2.84 μmoles g^−1^ DW in the transgenic lines. Content of 3C glucosinolate, 3-Methylsulfinylpropyl (glucoiberin, IBE) and 5C glucosinolate, 5-Methylsulfinyl (glucoalyssin, ALY) were found to be enhanced in most of the transgenic lines (Supplementary Table S5). There was no significant difference in the content of other glucosinolates in the seeds.

Sprouts are shown to accumulate highest levels of glucoraphanin^3^. Seven representative transgenic lines having seed glucoraphanin content >12.0 μmoles g^−1^ DW were selected and glucosinolate content in the four day old sprouts were analyzed using HPLC. Glucoraphanin content in the sprouts ranged from 18.39–35.13 μmoles g^−1^ DW against 0.28 μmoles g^−1^ DW of the wild-type, thereby showing more than 100 fold increases in the transgenic lines (Fig. 3c). Significant reduction in the major aliphatic glucosinolates like gluconapin and sinigrin were observed in the sprouts (Fig. 3d). Among other glucosinolates, glucoerucin and glucoalyssin were found increased significantly in the sprouts. The minor glucosinolate glucoiberin also showed significant enhancement in few transgenic lines (Fig. 3e). Progoitrin (PRO), an anti-nutritional glucosinolate found in remarkable amounts in sprouts remained unaltered in the transgenic lines (Fig. 3e). Overall, the total glucosinolates content showed a decline to ca. 58% in the sprouts of transgenic lines compared to the wild-type plants (Fig. 3f) suggesting key involvement of *GSL-ALK* locus towards controlling total glucosinolate content in *B. juncea*. Further, indolic glucosinolate pool remained almost unaltered in the transgenic lines (Fig. 3e).

### Molecular analysis of ALK-RNAi transgenic lines showed reduced levels of *GSL-ALK* gene transcripts

**Figure 4:**
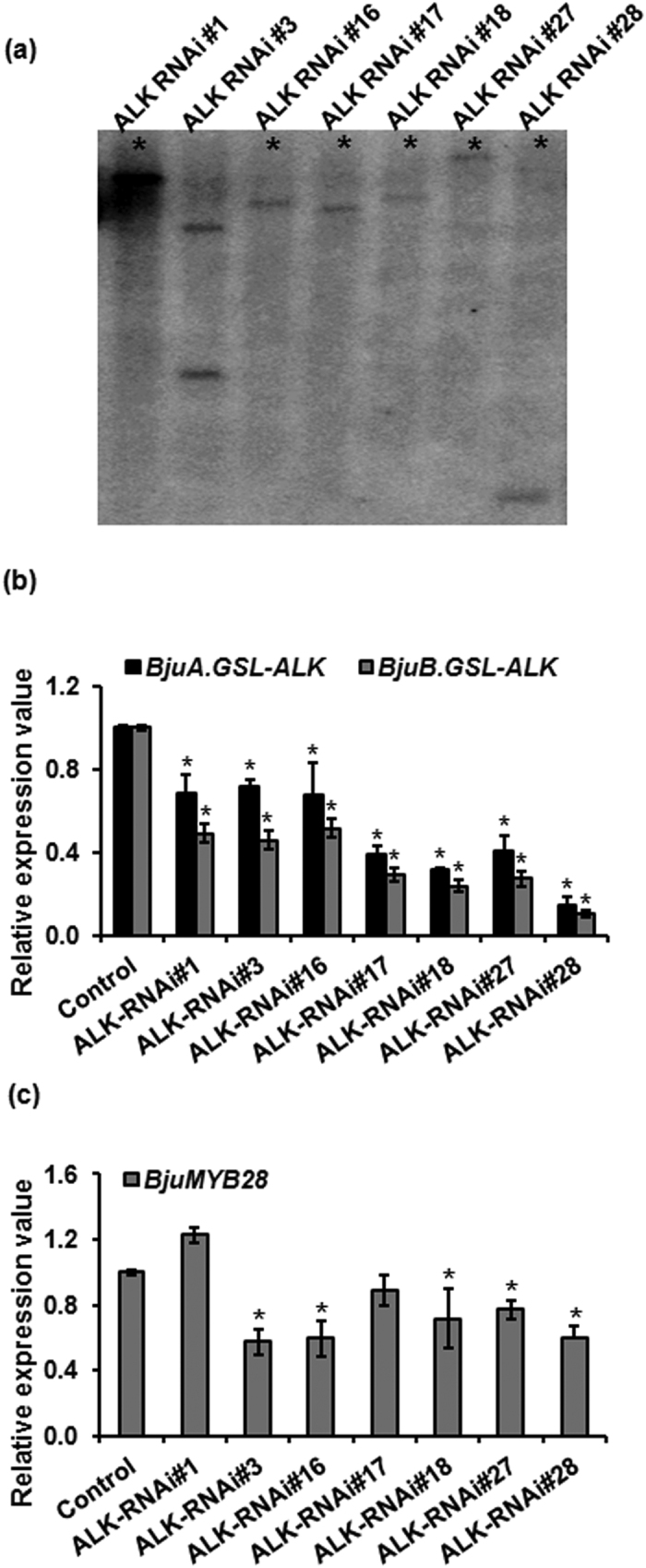
Molecular characterization of ALK-RNAi transgenic lines. (**a**) Southern blot analysis of selected ALK-RNAi lines. Asterisks represent single copy insertion of the T-DNA cassette. (**b**) Steady state mRNA levels of ALK-RNAi lines. qRT-PCR analysis of *GSL-ALK* genes was performed and the transcript accumulation was measured with reference to control (wild-type Varuna) set at 1. (**c**) Gene expression analysis of *BjuMYB28* genes in ALK-RNAi transgenic lines. Transcript level of *BjuMYB28* genes was estimated using qRT-PCR with primers conserved to *BjuMYB28* homologs. Asterisks on the top of bars indicate significant differences in gene expression compared to the wild-type control Varuna (set at 1) at *P* < 0.05, in Fishers LSD test determined by ANOVA.

Molecular characterization of the seven transgenic lines having highest levels of glucoraphanin content was carried out. T-DNA insertion number was ascertained using Southern blot and segregation analysis. Southern blot analysis of the T0 transgenic events showed stable integration of T-DNA cassette mostly in single copy (Fig. 4a). *Chi-*square analysis of T1 lines segregating for Basta resistance also confirmed single copy insertion of T-DNA cassette in most of the transgenic lines (Supplementary Table S6). However there seems no direct co-relation between the gene copy number and high glucoraphanin phenotype. For example, the high glucoraphanin accumulating line ALK-RNAi#3 was found to have T-DNA integration in more than one copy on southern blot. The variation in glucoraphanin content observed among the selected transgenic lines could be attributed to the position-effect mediated phenomenon.

The gene knock-down efficiency of ALK-RNAi construct was determined at transcriptional level. Steady state transcript accumulation, using real-time qRT-PCR analysis was carried out in the T1, Basta resistant progeny of each transgenic event. Primers, specific to *GSL-ALK* genes corresponding to the A- and B-sub-genome were used for transcript profiling. A significant reduction in the transcript levels of *GSL-ALK* gene homologs was observed compared to the control plants in all transgenic lines with both set of primers (Fig. 4b). The level of expression was found to be down regulated to variable extents, with a lowest value of at least five fold lower than that of the wild-type in ALK-RNAi lines. However, no direct relationship between the glucoraphanin content and transcript accumulation was observed. Since the total aliphatic glucosinolate content was also found to be lowered in the transgenic lines, expression of *MYB28* transcription factors which is the major positive regulator of aliphatic glucosinolate biosynthesis in *B. juncea*^27^ were also assayed in the transgenic lines. Interestingly, expression of *MYB28* homologs was also found to be altered across the ALK-RNAi transgenic lines with a significant down regulation observed in most of the transgenic lines (Fig. 4c).

### ALK-RNAi transgenic lines were stable for high glucoraphanin content and other seed quality traits in advance generations

**Figure 5:**
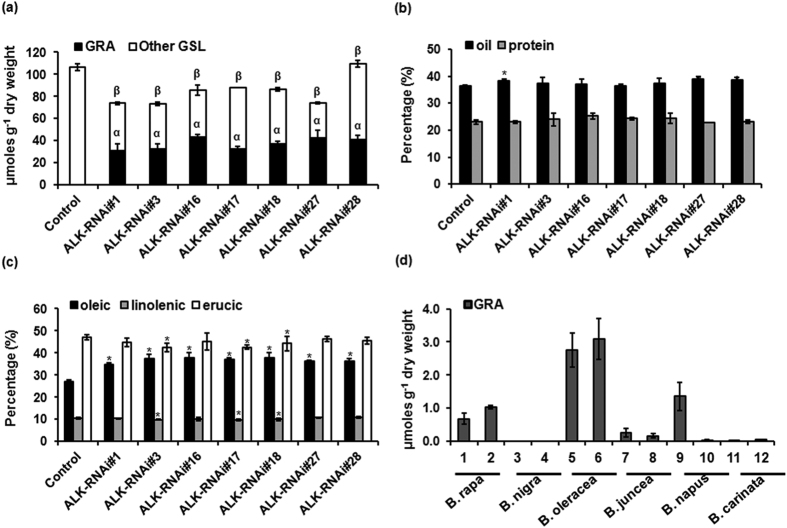
Characterization of ALK-RNAi transgenic lines for stability and seed quality traits. (**a**) Content of glucoraphanin and other glucosinolates in the seeds of ALK-RNAi lines in T2 generation as estimated by HPLC. (**b**) Total oil and protein content (**c**) Fatty acid composition (oleic, linolenic and erucic acid as estimated using Near Infra-red Reflectance Spectroscopy (NIRS). Average values of three independent measurements (n = 9) are represented along with standard deviation. Transformation host Varuna was used as control. Asterisk indicate significant difference at *P* < 0.001 in Fishers LSD as determined by ANOVA. (**d**) Screening of *Brassica* germplasm for glucoraphanin content. Glucoraphanin content (μmoles g^−1^ DW) in four day old sprouts of two cultivars each of six cultivated *Brassica* species was estimated by HPLC. (1. YID1, 2. Pusa Gold, 3. Sangam, 4. IC-257, 5. Pusa Sharad, 6. Palam Samridhi, 7. Heera, 8. Varuna, 9. HNS-9010, 10. NU-98, 11. BEC-218 and 12. CAR- 52.

Glucosinolate content were estimated in the T2 seeds of ALK-RNAi lines to check the stability of transgenic lines using HPLC. The glucoraphanin content was found as high as 43.11 μmoles g^−1^ DW in the seeds. Glucosinolate profile followed a similar trend as observed for the T1 seeds. However total glucosinolate content showed an enhancement due to the enrichment of glucoraphanin in the T2 seeds compared to T1 seeds (Fig. 5a). RNAi induced gene silencing often meet with off- target effects in crop plants. Since *B*. *juncea* is cultivated mainly as an oilseed crop, analysis of other seed quality parameters is also imperative. Total oil content, protein content and fatty acid compositions were analyzed in T2 seeds of the ALK-RNAi lines by NIRS. Total oil content of transgenic lines ranged from 36.27–39.02% compared to 36.46% in the wild-type, indicating lesser significant difference (Fig. 5b). Protein content is another important trait as far as *B. juncea* seed is concerned, as it is highly rich in protein and hence can be a substitute for soybean oil cake in the international market as cattle feed. When the total protein content of the transgenic lines was analyzed, there was no significant difference compared to the wild-type (Fig. 5b). Fatty acid compositions are important determinants of *Brassica* oil quality. Fatty acid compositions of the transgenic lines were also analyzed. Oleic acid (C18:1) which is known to be beneficial to humans was found to be significantly elevated in the ALK-RNAi transgenic lines. Oleic acid content of transgenic lines ranged from 34.69%–37.92% compared to 27.04% in the wild-type. Contents of other fatty acids namely, linolenic acid (C18:3) and erucic acid (C22:1) also showed significant enhancement in most of the transgenic lines compared to the wild-type which needs further investigation (Fig. 5c). The tested transgenic lines showed neither developmental nor phenotypic aberrations compared to the wild-type plants when grown under containment field conditions.

Further, we screened few *Brassica* germplasm to assess and compare the glucoraphanin content between the ALK-RNAi transgenic lines developed and commonly cultivated *Brassica* germplasm to check the superiority of these transgenic lines. Two representative cultivars from each of *Brassica* species commonly cultivated in India were screened for glucoraphanin content in 4-day old sprouts. There was only trace amount of glucoraphanin observed among *B. nigra, B. carinata* and *B. juncea* cultivars whereas *B. oleraceae, B. napus* and *B. rapa* showed somewhat higher amount of glucoraphanin content. *B. oleracea* cultivars Pusa Sharad (cauliflower) and Palam Samridhi (broccoli) showed maximum glucoraphanin content of around 2.75 and 3.08 μmoles g^−1^ DW respectively (Fig. 5d). Hence it can be assured that the ALK-RNAi transgenic lines developed in the current study can be a superior source for high glucoraphanin content up to 43.11 μmoles g^−1^ DW in the seeds. The sprouts are expected to have even higher levels of glucoraphanin.

### Susceptibility of ALK-RNAi lines towards *Sclerotinia sclerotiorum* infection

Glucosinolates are very important for plant fitness. When grown in field, the plant can undergo various biotic stresses in which fungal pathogens are one of the major challenges. The response of ALK-RNAi lines towards the stem rot pathogen *Sclerotinia sclerotiorum* was conducted since, not only the total glucosinolate content, but its profile also has altered in the transgenic lines. Four transgenic lines showing high glucoraphanin content were taken for analysis. The glucosinolate content and total phenol content of the leaf tissue were analyzed, as composition of both can alter pest status. Glucoraphanin content in leaves ranged from 6.27–22.11 μmoles g^−1^ DW of the leaf tissue (Table 1). Among other glucosinolates, the 5C glucosinolate, glucoalyssin showed a significant increase. There was no significant difference in the total phenol content of the transgenic lines compared to the wild-type plants (Table 1). Susceptibility of ALK-RNAi lines towards *S. sclerotiorum* was tested using disc assays using methanolic extracts of leaves. Interestingly, there was significant decrease in the mycelial diameter in the transgenic lines compared to the wild-type plants (Table 1; Supplementary Fig. S2). When compared to the mock control, effect of total glucosinolate in fungal growth retardation was also very evident. Effect of individual glucosinolates on fungal growth (diameter of mycelium) was predicted by linear regression analysis. Striking correlation was observed between the glucosinolate content and *S. sclerotiorum* growth. Mycelial diameter decreased significantly (*p* ≤ 0.05) with increase in content of glucoraphanin and glucoalyssin (Table 1). Mycelial diameter decreased by 0.061 cm and 0.378 cm with per unit increase in of glucoraphanin and glucoalyssin respectively. The content of all these glucosinolates were found to be accumulated in the transgenic lines at higher concentrations compared to the wild-type plants.

## Discussion

Brassicaceae is a large family of agriculturally important plants which consists of mustards, cabbages, broccoli, turnips, radish, kale, cresses, and their many relatives. Plants of this family are well known for the presence of the secondary metabolites called glucosinolates. Genetic analysis in *Arabidopsis* and *Brassica* crops has shown that QTLs like *GS-ELONG, GS-OX, GS-AOP* and *GS-OH* are important in determining glucosinolate content and its component variability^18^,^19^,^20^,^28^. *GSL-ELONG* locus controls chain length and is composed of *MAM1/MAM2* and *MAM3* genes. *FMO*_*GS-OX1-5*_is localized in *GS-OX* QTL and converts methylthioalkyl glucosinolates to the corresponding methylsulfinylalkyl glucosinolates^17^. QTL *GS-AOP* is composed of *GS-ALK* and *GS-OH* loci. *AOP2* is localized within *GS-ALK* whereas *AOP3* is localized within *GS-OH*^21^. These two loci are tightly linked and are responsible for converting methylsulfinylalkyl glucosinolates into alkenyl or hydroxyalkyl glucosinolates^16^,^20^. The presence or absence of either of the loci determines the type of glucosinolate produced in a particular species.

Glucoraphanin is the widely studied glucosinolate which has many health protective properties including cancer prevention. The occurrence of glucoraphanin is associated with *GSL-ALK* gene expression as evident by previous studies in *Arabidopsis*, *B. oleracea*, Chinese kale etc.^14^,^22^,^23^,^24^,^29^. However there is no Indian type *Brassica* accession known for high glucoraphanin content. In *B. juncea* the high level of accumulation of 4C glucosinolate, gluconapin which is the reaction product of glucoraphanin, indicate that the *GSL-ALK/AOP2* gene is fully functional in *B. juncea*. Enhancement of glucoraphanin through conventional breeding seems quite challenging as the conventional breeding strategies are not simpler in polyploid crops like *B. juncea* and *B*. *napus* where gene multiplicity and redundancy create complex genetic interactions. The presence of very high content of gluconapin also make the Indian *B. juncea* a promising candidate for achieving high glucoraphanin content through the silencing of *GSL-ALK* gene family. Hence, in the current study we isolated the *GSL-ALK* gene homologs from *B. juncea* and utilized it for the development of high glucoraphanin lines.

*B. juncea* is formed by the hybridization between the diploid species *B. rapa* and *B. nigra* which diverged 13–17 mya from the ancestral *Arabidopsis* lineage^26^. Whole genome triplication events followed by genome shrinkage shaped up the genome architecture of today’s *Brassica* species. Hence multiple copies of a given gene are expected which we could observe in the current study. Four *GSL-ALK* homologs, two of which representing A-sub-genome and other two representing B-sub-genome based on their sequence identity were isolated. Sequence analysis and phylogeny clearly showed that the *B. juncea*, *GSL-ALK* genes are the true orthologs of *AtAOP2* genes, which are required for the alkeylation of parent methylsulfinyl glucosinolates. In *B. oleracea* and *B. rapa* three copies of *AOP2* gene were identified^29^ whereas *B. napus* have up to nine members of *AOP*- like gene family^30^. We could isolate only two expressed members corresponding to A- sub-genome from *B. juncea*. The coding sequences of the four *GSL-ALK* homologs were found highly similar which shows that these genes are evolutionarily conserved. Similar level of conservation we could also observe while working with other glucosinolate genes like *MYB28*, *CYP83A1* and *GSL-ELONG*^27^,^31^. The coding sequences of *B. juncea GSL-ALK* genes encodes full-length protein, upon translation which further supports the assumption that these genes might be fully functional in *B. juncea*. This can further explain the presence of high amount of gluconapin and trace amount of glucoraphanin in *B. juncea* as well as the two progenitor genomes namely, *B. rapa* and *B. nigra*.

Knock-down of *GSL-ALK* gene have been shown to enhance glucoraphanin content in *B. napus* and Chinese kale^30^,^32^. RNAi based silencing of the *GSL-ALK* gene family in *B. napus* did not show significant alteration in the total glucosinolates content in the transgenic lines. However, the detrimental glucosinolate progoitrin was drastically reduced and glucoraphanin was increased to a high concentration in the seeds of F1 progeny of a cross between *B. napus* ALK-RNAi line and a double haploid line of high glucosinolate containing rapeseed^30^. The achievement of high glucoraphanin in F1 generation of *B. napus* makes the approach labour-intensive, time-consuming, and more importantly event-dependent, following a complex genetic control of glucosinolate trait in advance segregating generations. Moreover, gluconapin was found decreased only when the gene silencing was very strong. Similarly, in antisense mediated down regulation *of GSL-ALK* gene in Chinese kale, even though glucoraphanin content was increased, gluconapin content did not declin and surprisingly showed an enhancement^32^. But in our current study along with enhancement of glucoraphanin, we could observe a drastic decline in the concentrations of gluconapin and sinigrin as well as the total glucosinolate content in the ALK-RNAi lines in the initial T1 generation itself, which was also stable in advance T2 generation. Furthermore, the level of glucoraphanin accumulated in the selected *B. juncea* ALK-RNAi transgenic lines was even higher than the content of glucoraphanin reported in florets of Benefort’e^®^ broccoli^33^. Other 4C glucosinolate, glucoerucin which comes earlier in the pathway was found to be enhanced in the transgenic lines. The accumulation of 3C and 5C alkyl glucosinolate substrates namely, glucoiberin and glucoalyssin respectively in the ALK-RNAi lines further confirm that the RNAi construct developed in the study could effectively silence the target gene in *B. juncea*. Our study hence clearly demonstrates that RNAi based silencing of *GSL-ALK* gene homologs not only produces high amount of glucoraphanin in *B. juncea*, but also reduces un-desirable glucosinolates including gluconapin and sinigrin to a significantly lower level. The high glucoraphanin trait was found stable in the subsequent T2 generation with even higher levels of glucoraphanin accumulation.

Stable integration of T-DNA cassette is necessary to achieve consistent gene silencing phenomena in transgenic plants. Measurement of steady state mRNA levels of *GSL-ALK* gene in the transgenic lines showed significant down regulation of these transcripts reflecting the effectiveness of the RNAi construct against members of *GSL-ALK* gene family. Interestingly, in our study we observed a significant decline in total glucosinolates content also, suggesting that *GSL-ALK* gene might exert a regulatory role towards glucosinolate biosynthesis in *B. juncea*. It has been proposed that *AOP2* gene could be involved in a positive feedback regulation of glucosinolate biosynthesis^34^,^35^. Hence we measured the transcript levels of *MYB28* genes, the major positive transcriptional regulators of aliphatic glucosinolates and found that there was a significant reduction the transcript levels in most of the ALK-RNAi transgenic lines. Very recently it has been showed that, this feedback mechanism exists and is dependent on the glucosinolate transcriptional regulators MYB28 and MYB29 in *A. thaliana*^36^, which is somewhat similar to that observed in our study. However, the level of down regulation of *BjuMYB28* homologs was not very huge which could be due to the multiplicity of these genes. The highly complex glucosinolate biosynthesis in the polyploid *B. juncea* could be controlled by both MYB-dependent and independent regulatory networks, which needs detailed investigation.

Glucosinolates are primarily defense metabolites of the Brassicaceae plants. Altering the glucosinolate content and profile can alter the plant defense mechanism. In general, indolic glucosinolates are regarded as the critical determinants for pest and disease resistance whereas aliphatic glucosinolates play major role for the overall fitness of the plant^37^. However, contribution of individual glucosinolates towards particular pest and pathogen is not discovered in detail. In the current study, even though the indolic glucosinolates remained almost unaltered, the profile and content of aliphatic glucosinolates was altered. In *Arabidopsis* it has been shown that sulphoraphane, is an important defense arsenal against many pathogens *in vitro*^38^. Susceptibility of glucosinolate biosynthesis mutant, *gsm1-1* which was found to be largely deficient in two of the major antimicrobial compounds, including sulphoraphane was found responsive only to *Fusarium oxysporum* while showing no change in susceptibility towards other fungi like *Alternaria brassicicola*, *Plectosphaerella cucumerina*, *Botrytis cinerea*, *Peronospora parasitica*, and the bacteria *Erwinia carotovora* and *Pseudomonas syringae*^39^.

*Sclerotinia sclerotiorum* (Lib.) de Bary is a necrotrophic plant pathogen causing stem rot in *Brassica* species mainly in temperate areas of the world^40^,^41^. Recently the pathogen has emerged as a major pest in *B. juncea* in India also. Stem rot caused by *S. sclerotiorum* leads to severe yield loss in mustards^42^,^43^. The growth of *S. sclerotiorum* was found to be negatively correlated with glucoraphanin and glucoalyssin content. The resistance of these lines towards *S. sclerotiorum* infection can be attributed to high glucoraphanin and glucoalyssin in the transgenic lines. Studies by Stotz *et al.*^44^ suggested that accumulation of long chain glucosinolates is important for defense against *S. sclerotiorum* even though the role of sulphoraphane glucosinolates was not ruled out. Since indolic glucosinolate pool is not much affected, we do not expect much compromise on the defense response of the *B. juncea* ALK-RNAi transgenic lines. The susceptibility of the transgenic ALK-RNAi lines developed in the current research, against different pests/pathogens of *Brassica* species needs to be further studied to evaluate the potential for commercialization of the high glucoraphanin lines of *B. juncea*.

Overall, the study is an excellent example of translational research in *Brassica* crops. High glucoraphanin accumulation was achieved in all parts of the plant through constitutive down regulation of *GSL-ALK/AOP2* gene. The sprouts can be consumed in salads or leaves can be used for vegetable purpose, which can provide significant levels of the precursor anti-cancer glucosinolate, glucoraphanin to humans. Besides, industrial production of high amounts of glucoraphanin can also be achieved from seeds and sprouts of these high glucoraphanin lines of *B. juncea*. Hence in the global market, the commercial value of *B. juncea* can be raised not only as an oilseed but also as a health promoting food crop.

## Methods

### Plant materials and growth conditions

*B. juncea* (cv. Varuna) was grown in a growth chamber maintained at 10 h light/14 h dark cycle with a light intensity of 300 μmol m^−2^ s^−1^, temperature of 22 °C day/15 °C night, and relative humidity of 70%. *B. juncea* transgenic plants were selected by spraying herbicide Basta having active ingredient phosphinothricin (Bayer crop sciences, Mumbai, India) and maintained in the containment net-house facility available at NIPGR as per the guidelines of Department of Biotechnology, Government of India. For screening for glucoraphanin content in Indian grown *Brassicas*, *B. rapa* (YID1, Pusa Gold), *B. nigra* (Sangam, IC-257), *B. oleracea* (Pusa Sharad- cauliflower, Palam Samridhi- broccoli), *B*. *juncea* (Heera, Varuna), *B. napus* (HNS-9010, NU-98) and *B. carinata* (BEC-218, CAR-52) were used.

### Isolation of genomic and cDNA sequences of *GSL-ALK* homologs from *B. juncea*

The isolation of full-length genomic DNA sequences of the *GSL-ALK* genes was earlier reported by Bisht *et al.*^25^. The coding sequences of the *GSL-ALK* genes were amplified based on the primers designed using genomic sequences and PCR amplifications were carried out in *B. juncea* (cv. Varuna), *B. nigra* (cv. IC-257) and *B. rapa* (cv. YID1). RT- PCR products were cloned into pGEM-T Easy cloning vector (Promega, Madison, Wisconsin, USA) and sequenced to check correctness of the sequence. Progenitor genomes of *BjuGSL-ALK* genes were assigned based on the sequence comparison with the homologs obtained from *B. nigra* and *B. rapa*. All sequence analysis was performed using Lasergene core suite (DNASTAR, Madison, Wisconsin, USA). A list of primers used in the current study is summarized in Supplementary Table S7.

### Phylogenetic analysis and estimation of divergence time

Coding sequences *AOP* genes of *A. thaliana* ecotype Columbia (At4g03070, At4g03050), *B. rapa* (Bra018992, Bra034180, Bra034181, Bra034182, Bra000847, Bra000848, Bra018521,) *B. oleracea* (Bol030626, Bol030626) were retrieved from BRAD database (http://brassicadb.org/brad) whereas sequence of *B. oleracea* (AY044424.1, AY044425.1), *A. thaliana* ecotype Cape Verdi Islands (Cvi) (AF417859, AF417859) and Columbia (At4g03060) were retrieved from NCBI and TAIR databases. Multiple sequence alignment of the full-length coding sequences was generated using ClustalW and the evolutionary tree was constructed using Maximum Likelihood method in MEGA5^45^.

To determine the divergence time of *AOP* homologs, coding DNA sequences from *Brassica* species were aligned using ClustalW. Synonymous (Ks) and non-synonymous (Ka) base substitution were calculated by DnaSP v5 software^46^. The divergence time of *AOP* genes was calculated using the equation, divergence time in MYA, T = Ks/(2×[1.5 × 10^−8^] where 1.5 × 10^−8^ substitution per site per year is the synonymous mutation rate reported for *Brassica* genus^47^.

### Generation of ALK-RNAi transformation construct and knock-down lines in *B. juncea*

Binary vector pPZP200lox:bar having 35Sde-*bar*-ocspA fragment conferring resistance to herbicide Basta was used for generating transgenic plants. Approximately 325 bp fragment (949 bp downstream of ATG start codon) of *BjuGSL-ALK* genes was amplified and cloned in both sense and antisense orientation on either side of *BjuMYB28* intron to generate an intron spliced hairpin RNAi cassette^12^. The Exon(sense)-int2-Exon(antisense)-35SpA cassette driven by CaMV35S promoter was cloned into the binary vector pPZP200lox:35Sde-*bar*-ocspA to create the final transformation cassette. The cassette was mobilized into *A. tumefaciens* strain GV3101 and later into *B. juncea* cv. Varuna using the protocol described earlier^13^. Primers used for the construct design are provided in Supplementary Table S7.

### Glucosinolate analysis using HPLC

The seeds, sprouts and leaves of transgenic lines were analyzed for their individual glucosinolate contents using HPLC as per the protocols described earlier^12^. p-hydroxylbenzyl glucosinolate (Sinalbin), purified from *Sinapis alba* (kindly provided by Dr. Michael Reichelt, Max Plank Institute of Chemical Ecology, Jena Germany) was used as the internal standard. Concentrations of individual glucosinolate were calculated relative to the area of the internal standard peak applying their relative response factors and expressed in μmoles g^−1^ dry weight (DW) of the tissue. Data from three independent experiments were averaged and standard deviation was calculated. Test of significance were carried out using one way ANOVA following Fishers LSD.

### Estimation of total protein, oil content and fatty acid composition

Total protein, oil content and fatty acid (oleic acid, linoleic acid, erucic acid) composition of transgenic seeds were determined using Near Infrared Reflectance Spectroscopy^48^ (NIRS, DS-2500, FOSS, Denmark) as per manufacturer’s instructions. Briefly, intact *B. juncea* seed samples were placed in the NIRS sample holder (3 cm diameter round cell) until it was three-fourths full, and their spectra were registered as an individual file, in the range from 400 to 2500 nm, at 0.5 nm interval. Using the program Global Calibration Development software (WINISI III, Infrasoft International, LLC, Port Matilda, PA), different calibration equations were developed on the calibration set (n ~ 550) having variable ranges of these seed quality parameters. Calibration equations were then validated on known seed samples, and subsequently used for the experimental test samples. Seeds of wild-type Varuna were used as control. Statistical analyses were conducted using one-way ANOVA applying Fishers LSD test of significance.

### Southern blot analysis for identification of single copy insertions

Single copy insertions of the T-DNA cassette were identified by Southern blot analysis according to the protocol described earlier^12^. Briefly, genomic DNA was isolated using CTAB method and ca. 20 μg of purified genomic DNA digested with *EcoRV* was used for blotting. Selection marker gene, *bar* (390 bp) was used as the probe which was labelled with dCTP [^32^Pα] using Amersham Megaprime DNA labelling systems (GE Healthcare, Littlechalfont, Buckinghamshire, UK) according to the manufacturer’s instructions.

### Real-time quantitative RT-PCR (qRT-PCR) analysis

The transcript levels of *GSL-ALK* genes were analyzed using quantitative real-time RT-PCR in ABI 7900HT Fast Real-time PCR machine (Applied Biosystems, Foster city, CA, USA) following SYBR green protocol. Total RNA was isolated using Spectrum Total RNA Isolation Kit (Sigma-Aldrich, St. Louis, Missouri, USA). Two micrograms each of total RNA were reverse-transcribed using high capacity first strand cDNA synthesis kit (Applied Biosystems, Foster city, CA, USA) according to manufacturer’s instructions. The *BjuACTIN2* gene was used as endogenous control^49^. Three independent sets of experiments were performed with three technical replicates each to finalize the data. Primers used for qRT-PCR analysis are tabulated in Supplementary Table S7.

### Estimation of total phenolic content

Total phenolic content were estimated using Folin-ciocalteu (FC) reagent^50^,^51^. Exactly 100 mg lyophilized plant tissues were extracted twice with 2 ml each of hot 70% methanol. The extract was diluted 10 fold and used for the assay. For the assay 250 μl of the diluted sample was aliquoted into assay vials in duplicates and 1.25 ml of FC reagent (10%) was added to each vial. To this one ml of Na_2_CO_3_ (7.5%) was added and the contents were mixed thoroughly by vortexing. The reaction was allowed to stand for one hour at room temperature and readings were taken in a spectrophotometer (SmartSpec Plus, Bio-Rad laboratories) at 765 nm. Gallic acid was used as the standard and the total phenolics content was expressed as gallic acid equivalent (GAE) 100 mg^−1^ dry weight of the tissue.

### Fungal infectivity assays

Stem rot pathogen of *B. juncea, S. sclerotiorum* (Delhi isolate-I, kindly provided by Dr. Jagreet Kaur, University of Delhi, India) was used for the susceptibility assays. Disc assay earlier described by Sotelo *et al.*^40^ was used with minor modifications. PDA plates (90 mm) were prepared with 100 μl each of leaf methanolic extracts of 30 day old transgenic (T1) lines and the control plants. Fungal pathogen was grown in separate PDA plates for three days and 6 mm disc of the PDA medium containing the actively growing mycelium was placed on the centre of each plates. PDA plate with 70% methanol was used as negative control (mock). Plates were incubated at 22 °C and growth diameter of mycelium (in cm) was measured after three days. The experiment was repeated four times with three technical replicates each time. Glucosinolates profiles of the tested plant samples were analyzed by HPLC. To examine the relationship between glucosinolates content and mycelial diameter, the data were fitted with simple linear model (*y* = *a* + *bx*). Linear regression analysis was performed in SPSS software package v.17.0 for Windows (SPSS Inc. released in 2008), by considering glucosinolates content as independent variable and mycelial diameter as dependent variable.

## Additional Information

**How to cite this article**: Augustine, R. and Bisht, N. C. Biofortification of oilseed *Brassica juncea* with the anti-cancer compound glucoraphanin by suppressing *GSL-ALK* gene family. *Sci. Rep.* **5**, 18005; doi: 10.1038/srep18005 (2015).

## Supplementary Material

Supplementary Information

## Figures and Tables

**Figure 1:**
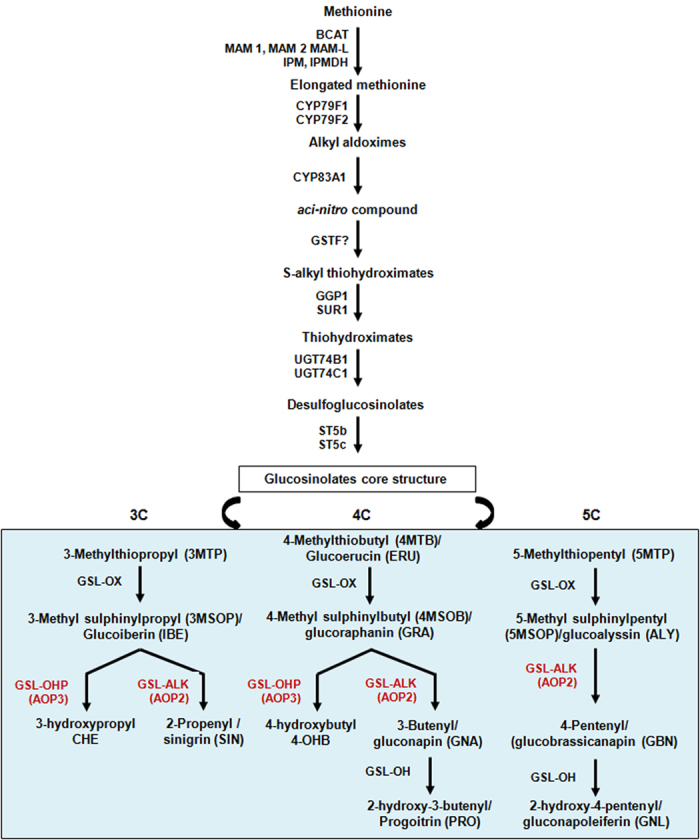
General scheme for aliphatic glucosinolate biosynthesis. Precursor amino acid (here methionine) undergoes a series of reactions to form the glucosinolate core structure. The core structure further undergoes modifications of its side chain generating diverse glucosinolate structures. The side chain modification step is represented in box. Enzymes catalyzing specific steps are also indicated. Chemical name, trivial name and abbreviations of individual glucosinolates are given in parenthesis. C3, C4 and C5 represent glucosinolates with 3, 4 and 5 carbon chain respectively in the core structure.

**Table 1:** Glucosinolate (GSL in μmoles g^−1^ DW), total phenol content (TPC in GAE 100 mg^−1^ tissue) and mycelial growth (cm) of *S.* s*clerotiorum* in methanolic extracts of ALK-RNAi lines.

Line	IBE	GRA	SIN	ALY	GNA	GBN	Indolic GSL	Total GSL	TPC	Mycelial Diameter
Mock	—	—	—	—	—	—	—	—	—	9.00 ± 0.24
Control	0.06 ± 0.02	0.07 ± 0.05	8.67 ± 0.18	0.10 ± 0.01	57.12 ± 1.82	3.47 ± 0.02	1.02 ± 0.31	70.76 ± 2.11	0.93 ± 0.09	6.32 ± 0.25^a^
ALK-RNAi#1	0.44 ± 0.02	6.27 ± 0.43^*^	3.06 ± 0.09	1.62 ± 0.09	16.02 ± 1.64	0.17 ± 0.10	0.47 ± 0.13^*^	28.26 ± 1.81^*^	0.97 ± 0.08	5.61 ± 0.13^ab^
ALK-RNAi#3	1.49 ± 0.20^*^	16.08 ± 1.96^*^	1.59 ± 0.15	2.66 ± 0.19^*^	6.74 ± 0.55	0.63 ± 0.39	1.08 ± 0.34	30.53 ± 3.66^*^	0.90 ± 0.16	5.72 ± 0.42^ab^
ALK-RNAi#16	2.01 ± 0.16^*^	18.22 ± 1.78^*^	2.04 ± 0.45	3.09 ± 0.19^*^	6.23 ± 0.35	0.13 ± 0.38	1.19 ± 0.06	32.97 ± 2.46^*^	0.70 ± 0.01	5.33 ± 0.34^ab^
ALK-RNAi#27	2.31 ± 0.07^*^	22.11 ± 0.31^*^	1.68 ± 0.12	4.45 ± 0.15^*^	6.85 ± 1.52	0.21 ± 0.26	0.78 ± 0.40	38.54 ± 1.26^*^	0.73 ± 0.05	5.43 ± 0.31^ab^

Concentration of individual glucosinolates and total phenol content in the leaves of ALK-RNAi lines (T1) used for fungal infectivity assay is given. Asterisk indicates significant increase in glucosinolate compared to the wild-type control. Letter^a^ on top indicate significant difference in mycelial diameter compared to mock (methanol) and letters^ab^ represent significant difference from both mock and wild-type control. Statistical analysis was conducted using one-way ANOVA following Fishers LSD test of significance at *P* < 0.05.
