# Supplementary material for: Biofortification of oilseed Brassica juncea with the anti-cancer compound glucoraphanin by suppressing GSL-ALK gene family
Source: Sci Rep. 2015 Dec 10;5:18005. doi: 10.1038/srep18005 (PMC4997087; doi:10.1038/srep18005)
Supplement: Supplementary Information [file srep18005-s1.doc]

**Title:** Biofortification of oilseed *Brassica juncea* with the anti-cancer compound glucoraphanin by suppressing *GSL-ALK* gene family

**Authors:** Rehna Augustine and Naveen C. Bisht^*^

**Supplementary Information**

***Table S1.*** *Comparison of gene structure of GSL-ALK gene homologs from* B. juncea.

| **Gene** | **Progenitor genome** | **Gene (bp)** | **CDS (bp)** | **No. of exons (position in bp)** | **No. of Introns (length in bp)** | **Protein**  **(aa)** | **% identity to *AtAOP2* (Cvi)** |
| --- | --- | --- | --- | --- | --- | --- | --- |
| ***BjuA.GSL-ALK-1*** | AA | 1809 | 1323 | 3 (1-372, 659-1349, 1548-1809) | 2 (294,167) | 441 | 75.6 |
| ***BjuA.GSL-ALK-2*** | AA | 1806 | 1320 | 3 (1-372, 659-1349, 1548-1809) | 2 (294,167) | 440 | 75.5 |
| ***BjuB.GSL-ALK-1*** | BB | 1809 | 1323 | 3 (1-372, 659-1349, 1548-1809) | 2 (288,200) | 441 | 75.3 |
| ***BjuB.GSL-ALK-2*** | BB | 1806 | 1320 | 3 (1-372, 659-1349, 1548-1809) | 2 (288,200) | 440 | 74.8 |

**Table S2.** Identity percentage (%) of *BjuGSL-ALK* coding sequences with that of *AtAOP2* (*A. thaliana* Cvi.) The percentage identity was estimated using Lasergene core suite (DNASTAR, Madison, Wisconsin, USA).

| ***BjuA.GSLALK-1*** | ***BjuA.GSLALK-2*** | ***BjuB.GSLALK-1*** | ***BjuB.GSLALK-2*** |  |
| --- | --- | --- | --- | --- |
| 75.6 | 75.5 | 75.3 | 74.8 | **AF417858 (Cvi)** |
|  | 99.6 | 92.0 | 90.8 | ***BjuA.GSLALK-1*** |
|  |  | 92.0 | 91.1 | ***BjuA.GSLALK-2*** |
|  |  |  | 98.5 | ***BjuB.GSLALK-1*** |
|  |  |  |  | ***BjuB.GSLALK-2*** |

**Table S3.** Amino acid sequence identity (%) of deduced BjuGSL-ALK proteins with that of AtAOP2 (*A. thaliana* Cvi.) The percentage identity was estimated using Lasergene core suite (DNASTAR, Madison, Wisconsin, USA)

| **BjuA.GSLALK-1** | **BjuA.GSLALK-2** | **BjuB.GSLALK-1** | **BjuB.GSLALK-2** |  |
| --- | --- | --- | --- | --- |
| 59.6 | 59.4 | 58.9 | 58.2 | **AF417858 (Cvi)** |
|  | 99.3 | 89.1 | 87.1 | **BjuA.GSLALK-1** |
|  |  | 89.1 | 87.7 | **BjuA.GSLALK-2** |
|  |  |  | 98.0 | **BjuB.GSLALK-1** |
|  |  |  |  | **BjuB.GSLALK-2** |

**Table S4.** Divergence time analysis of *GSL-ALK* genes based on synonymous nucleotide substitutions using DnaSP v5 software.

| **Gene** | **Ks** | **Ka** | **Divergence time (mya)** |
| --- | --- | --- | --- |
| **Between *A. thaliana* Cvi (AF417858) and *B. juncea* (AABB genome)** | | | |
| BjuA.GSLALK-1 | 0.44 | 0.29 | 14.82 |
| BjuA.GSLALK-2 | 0.44 | 0.29 | 14.78 |
| BjuB.GSLALK-1 | 0.48 | 0.29 | 16.01 |
| BjuB.GSLALK-2 | 0.48 | 0.30 | 16.05 |
| **Between *A. thaliana* Cvi (AF417858) and other orthologs of*AOP2* clade** | | | |
| Bra000848 | 0.68 | 0.47 | 22.88 |
| Bra018521 | 0.45 | 0.29 | 15.08 |
| Bra034180 | 0.44 | 0.29 | 14.73 |
| AY044425.1 | 0.44 | 0.29 | 14.76 |
| AY044424.1 | 0.45 | 0.28 | 14.97 |

| **Lines** | **IBE** | **GRA** | **SIN** | **GNL** | **GNA** | **ALY** | **GBN** | **ERU** | **Total aliphatic** |
| --- | --- | --- | --- | --- | --- | --- | --- | --- | --- |
| ALK1 | 1.92 | 12.54 | 3.66 | 0.41 | 13.98 | 2.74 | 0.00 | 0.75 | **36.00** |
| ALK2 | 2.45 | 12.85 | 6.26 | 0.41 | 23.83 | 2.73 | 0.16 | 1.21 | **49.90** |
| ALK3 | 4.73 | 19.82 | 3.87 | 0.52 | 15.47 | 3.43 | 0.09 | 1.76 | **49.69** |
| ALK4 | 1.61 | 9.75 | 6.23 | 0.49 | 25.28 | 2.24 | 0.16 | 0.90 | **46.65** |
| ALK5 | 0.53 | 5.95 | 6.90 | 0.30 | 29.40 | 2.31 | 0.31 | 0.52 | **46.21** |
| ALK6 | 0.00 | 0.11 | 12.31 | 0.00 | 48.89 | 1.81 | 0.54 | 0.00 | **63.65** |
| ALK7 | 3.66 | 19.98 | 6.08 | 0.66 | 23.32 | 2.37 | 0.10 | 1.80 | **57.97** |
| ALK8 | 0.29 | 4.46 | 6.15 | 0.23 | 28.27 | 1.29 | 0.34 | 0.35 | **41.38** |
| ALK9 | 1.68 | 11.55 | 10.36 | 0.76 | 42.75 | 1.63 | 0.39 | 1.95 | **71.08** |
| ALK10 | 1.62 | 9.73 | 6.54 | 0.36 | 24.78 | 1.50 | 0.00 | 1.79 | **46.33** |
| ALK11 | 4.68 | 24.72 | 8.81 | 0.77 | 32.33 | 2.17 | 0.00 | 2.84 | **76.30** |
| ALK12 | 0.00 | 2.30 | 7.10 | 0.20 | 33.73 | 1.54 | 0.42 | 0.19 | **45.48** |
| ALK13 | 0.53 | 6.59 | 6.18 | 0.36 | 23.48 | 1.48 | 0.09 | 0.51 | **39.22** |
| ALK14 | 6.62 | 12.10 | 5.62 | 0.15 | 9.30 | 1.02 | 0.00 | 1.07 | **35.87** |
| ALK15 | 0.22 | 4.73 | 6.71 | 0.26 | 29.91 | 1.14 | 0.26 | 0.29 | **43.53** |
| ALK16 | 3.74 | 17.83 | 3.11 | 0.50 | 11.81 | 1.55 | 0.00 | 1.49 | **40.04** |
| ALK17 | 2.88 | 15.47 | 5.57 | 0.54 | 20.18 | 1.10 | 0.00 | 1.55 | **47.29** |
| ALK18 | 2.69 | 13.99 | 2.78 | 0.43 | 11.58 | 1.14 | 0.00 | 1.02 | **33.62** |
| ALK20 | 0.00 | 0.03 | 12.86 | 0.03 | 50.80 | 0.66 | 0.75 | 0.00 | **65.13** |
| ALK22 | 2.65 | 13.77 | 2.93 | 0.41 | 10.37 | 0.00 | 0.00 | 1.81 | **31.95** |
| ALK23 | 0.00 | 1.57 | 4.68 | 2.70 | 20.49 | 0.00 | 3.21 | 0.37 | **33.03** |
| ALK25 | 1.81 | 11.43 | 2.96 | 0.43 | 11.68 | 2.48 | 0.00 | 1.15 | **31.95** |
| ALK27 | 3.56 | 16.18 | 3.17 | 0.46 | 12.01 | 2.29 | 0.00 | 1.97 | **39.63** |
| ALK28 | 2.36 | 13.08 | 2.74 | 0.44 | 10.84 | 1.93 | 0.00 | 1.37 | **32.75** |
| ALK29 | 0.00 | 0.61 | 8.77 | 0.08 | 42.19 | 1.30 | 0.68 | 0.00 | **53.62** |
| ALK30 | 0.10 | 2.21 | 8.38 | 0.15 | 39.73 | 1.78 | 0.45 | 0.13 | **52.92** |
| ALK31 | 0.97 | 9.50 | 6.14 | 0.56 | 29.16 | 0.86 | 0.00 | 1.43 | **48.63** |
| ALK32 | 0.00 | 0.00 | 6.43 | 0.00 | 34.23 | 0.83 | 0.61 | 0.00 | **42.10** |
| ALK33 | 2.57 | 14.48 | 6.65 | 0.56 | 28.24 | 1.17 | 0.00 | 2.60 | **56.27** |
| ALK34 | 0.00 | 1.11 | 8.58 | 0.13 | 39.55 | 1.21 | 0.53 | 0.00 | **51.10** |
| ALK37 | 0.80 | 5.23 | 1.94 | 0.22 | 7.98 | 0.34 | 0.00 | 1.13 | **17.65** |
| Control | 0.20 | 0.01 | 21.08 | 0.10 | 93.55 | 0.58 | 0.99 | 0.04 | **119.59** |

**Table S5**. Average aliphatic glucosinolate content (μmoles g^-1^ DW) in T1 seeds of ALK-RNAi lines as estimated by HPLC.

IBE (3-Methylsulfinylpropyl glucosinolate, Glucoiberin); GRA (4-Methylsulfinylalkyl glucosinolate, glucoraphanin); SIN (2-Propenyl glucosinolate, Sinigrin); GNL (2-Hydroxy-4-pentenyl glucosinolate, Gluconapoleiferin); GNA (3-butenyl glucosinolate, Gluconapin); ALY (5-Methylsulfinyl, Glucoalyssin); GBN (4-Pentenyl glucosinolate, Glucobrassicanapin); ERU (4-Methylthiobutyl, Glucoerucin)

**Table S6. *Chi*- square analysis and deduced copy number of T-DNA insertion in transgenic ALK(RNAi) lines. *Chi*-square value less than 3.84 indicates single copy insertion.**

| **Transgenic line** | ***Chi*-square** | **Copy no.** |
| --- | --- | --- |
| ALK(RNAi)#1 | 0.55 | 1 |
| ALK(RNAi)#3 | 0.00 | 1 |
| ALK(RNAi)#16 | 0.73 | 1 |
| ALK(RNAi)#17 | 4.30 | >1 |
| ALK(RNAi)#18 | 0.01 | 1 |
| ALK(RNAi)#27 | 0.11 | 1 |
| ALK(RNAi)#28 | 0.29 | 1 |

**Table S7. List of primers used in the study.**

| **Primer** | **Sequence 5'-3'** | **Description** |
| --- | --- | --- |
| GSL-ALK FP | ATGGGTTCAGACAGTACTCCTC | Forward primer for amplifying *GSLALK* homologs |
| GSL-ALK RP | TTATGCTCCAGAGACGGCACAATAAGC | Reverse primer for amplifying *GSLALK* homologs |
| GANS FP | CGTGTCTCCGAAACCCTACATG | FP for realtime PCR of *BjuB.GSLALK-1* |
| GANS RP | CAGTCGCATACGGTAATTCTTCAA | RP for realtime PCR of *BjuB.GSLALK-2* |
| GARS FP | ACGTGTCTCCGAAACCGTACAC | FP for realtime PCR of *BjuA.GSLALK-1* |
| GARS RP | TCTCTATGACCATTCTTCTCACCA | FP for realtime PCR of *BjuA.GSLALK-2* |
| ALKRNAi FPS | ACTCGAGAACCAAAGATGAAAAGTGGAT | FP for cloning sense fragment of *GSLALK* for RNAi construct |
| ALKRNAi RPS | ACCATGGCAGAGACGGCACAATAAGCGTG | RP for cloning sense fragment of *GSLALK* for RNAi construct |
| ALKRNAi FPAS | ATCTAGAAACCAAAGATGAAAAGTGGAT | FP for cloning antisense fragment of *GSLALK* for RNAi construct |
| ALKRNAi RPAS | ACCCGGGCAGAGACGGCACAATAAGCGTG | RP for cloning antisense fragment of *GSLALK* for RNAi construct |
| Bjactin FP | CTTCTTACCGAGGCTCCTCT | Real-time endogenous control FP |
| Bjactin RP | AAGGATCTTCATGAGGTAATCAGT | Real-time endogenous control RP |


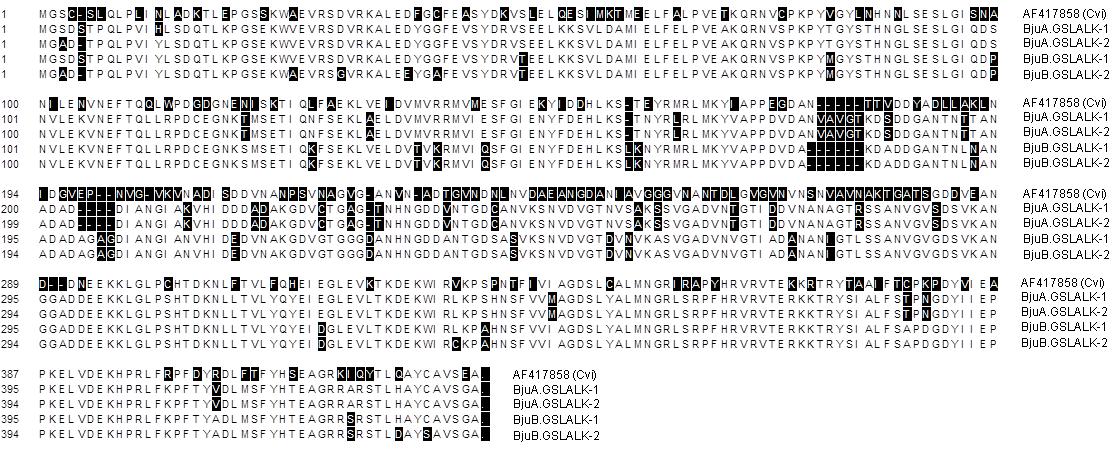


**Fig. S1.** Amino acid sequence alignment of GSL-ALK proteins isolated from *Brassica juncea* along with *A. thaliana* AOP2 (Cvi, AF417858). Residues differing from consensus are shaded black.


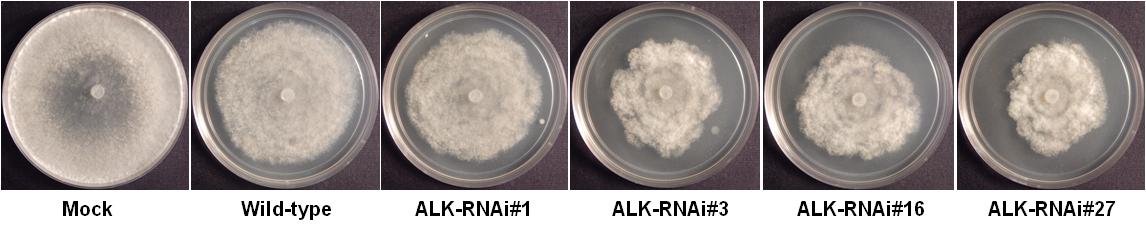


**Fig. S2.** Growth pattern of *S. sclerotiorum* in PDA plates containing methanolic extracts of ALK- RNAi lines. The stem rot pathogen *S. sclerotiorum* (Delhi Isolate-I) was grown in PDA plates containing leaf methanolic extracts of selected ALK-RNAi lines. PDA discs of 6 mm diameter containing the actively growing mycelia with uniform growth were placed on the centre of the test plates. Diluted methanol (70%) was used as mock. The mycelial diameter was recorded after three days of inoculation. A representative figure is shown.
